# The Self-Reference Effect on Memory Is *Not* Diminished in Autism: Three Studies of Incidental and Explicit Self-Referential Recognition Memory in Autistic and Neurotypical Adults and Adolescents

**DOI:** 10.1037/abn0000467

**Published:** 2019-10-31

**Authors:** Sophie E. Lind, David M. Williams, Toby Nicholson, Catherine Grainger, Peter Carruthers

**Affiliations:** 1 Department of Psychology, City, University of London; 2 School of Psychology, University of Kent; 3 Department of Psychology, University of Stirling; 4 Department of Philosophy, University of Maryland, College Park

**Keywords:** autism spectrum disorder, recognition memory, self-awareness, self-bias, self-reference effect

## Abstract

Three experiments investigated the extent to which (a) individuals with autism show a self-reference effect (i.e., better memory for self-relevant information), and (b) the size of the self-reference effect is associated with autism traits. Participants studied trait adjectives in relation to their own name (self-referent) or a celebrity’s name (other-referent) under explicit and incidental/implicit encoding conditions. Explicit encoding involved judging whether the adjectives applied to self or other (denoted by proper names). Implicit encoding involved judging whether the adjectives were presented to the right or left of one’s own or a celebrity’s name. Recognition memory for the adjectives was tested using a yes/no procedure. Experiment 1 (individual differences; *N* = 257 neurotypical adults) employed the Autism-spectrum Quotient as a measure of autistic traits. Experiments 2 (*n* = 60) and 3 (*n* = 52) involved case-control designs with closely matched groups of autistic and neurotypical adults and children/adolescents, respectively. Autistic traits were measured using the Autism-spectrum Quotient and Social Responsiveness Scale, respectively. In all experiments, a significant self-reference effect was observed in both explicit and implicit encoding conditions. Most importantly, however, there was (a) no significant relation between size of the self-reference effect and number of autistic traits (Experiments 1, 2, and 3), and (b) no significant difference in the size of the self-reference effect between autistic and neurotypical participants (Experiments 2 and 3). In these respects, Bayesian analyses consistently suggested that the data supported the null hypothesis. These results challenge the notion that subjective or objective self-awareness are impaired in autism.

## General Scientific Summary

It is generally easier to remember information that is relevant to oneself than to remember other kinds of information. This is known as the “self-reference effect.” Previously, it has been claimed that people with autism show a reduced self-reference effect (implying diminished self-awareness), but this study provides robust evidence that people with autism are, in fact, just as susceptible to this effect as neurotypical people.

The study of self has a long history in psychology and philosophy. One prominent distinction is made between different levels of self-awareness. On the one hand, the self is the subject of consciousness—an entity that experiences and acts on the world. This “subjective” level was referred to by James (1890) as the “I” and involves only a first-order representation of self. On the other hand, the self can also be thought about; it can be the object of thought/consciousness, as well as the subject. This “objective” level was referred to by James (1890) as the “Me” and necessarily involves a second-order representation of self. Crucially, it has long been argued that memory and the self are inextricably linked, and that self-representation influences cognition more generally in specific ways (e.g., Conway, 2005; Hume, 1739/2003; James, 1890; Locke, 1690/1995; Prebble, Addis, & Tippett, 2013; Wilson & Ross, 2003). One of the clearest empirical demonstrations of this is the so-called “self-reference effect,” whereby information encoded in relation to the self has a memory advantage over information encoded in other ways (Rogers, Kuiper, & Kirker, 1977). There is extensive and robust evidence for this effect, and it occurs across a range of memory tasks and encoding conditions (Symons & Johnson, 1997).

In classic self-referential trait memory tasks (e.g., Kuiper, 1982), participants are presented with personality trait adjectives (e.g., patient, tenacious, arrogant) usually under two encoding conditions: self-referential and other-person-referential (typically a well-known celebrity). In the self-referential condition, the trait adjective must be processed in relation to the self: for example, by asking, “Is [participant’s name] patient?” In the other-person-referential condition, the adjective must be processed in relation to another person: for example, by asking, “Is Teresa May tenacious?” Numerous studies have shown that recall and recognition of trait labels judged in relation to self is superior to recall and recognition of trait labels judged in relation to another person (Symons & Johnson, 1997). This trait evaluation memory paradigm is the most widely used measure of self-reference in the literature and has been pivotal to the development of theories about self-referential cognition for nearly 40 years (Conway & Dewhurst, 1995; Klein & Kihlstrom, 1986; Klein & Loftus, 1988; Symons & Johnson, 1997). The effect is remarkably robust and meta-analytic results suggest that self-reference is the most efficient encoding mechanism for promoting memory (Symons & Johnson, 1997). The traditional self-bias on the trait memory paradigm is associated with activity in prefrontal cortex (particularly the dorsal medial region; e.g., Sui & Humphreys, 2017; Turk, Van Bussel, Waiter, & Macrae, 2011), a region of the brain considered to underpin/contribute to conscious reflection on and evaluation of oneself (Schmitz & Johnson, 2007).

The self-reference effect has traditionally been thought to result from a high-level cognitive process that involves “deep” elaborative encoding of information (Craik & Tulving, 1975): By becoming objectively self-aware during the encoding phase on self-referential trials, one’s explicit, second-order self-representation (the “Me”) scaffolds encoding of the trait word and leads to better recall/recognition during the memory test phase. However, more recently the self-reference effect has been proposed to originate in an implicit fashion (e.g., Sui & Humphreys, 2015; Cunningham, Brebner, Quinn, & Turk, 2014), via a subjective, first-order representation of self (the “I”). According to this view, low-level self-awareness biases attention, perception, memory, and motor planning in a relatively automatic manner, without requiring any explicit thought about oneself. Turk, Cunningham, and Macrae (2008; also see Cunningham et al., 2014) explored this by creating an “incidental” encoding condition of the trait memory paradigm, in which participants were asked to judge whether trait adjectives appeared above or below their name or a celebrity’s name. Thus, participants were required merely to make a spatial judgment that involved only a superficial form of processing (iconic memory), as opposed to deep semantic processing of/thought about self-related information. Yet, during a subsequent recognition phase, participants showed a significant self-reference effect in this incidental condition as well as during the classic evaluative processing condition (although the size of the self-bias was significantly larger in the explicit condition).

The research discussed thus far has major implications for our understanding of autism spectrum disorder (ASD). ASD has long been identified as involving an atypical awareness of self (e.g., Hobson, 1990; Frith, 2003; Lombardo & Baron-Cohen, 2010), and several hypotheses concerning its relation to its behavioral phenotype have been proposed. For example, Hobson (2010) has argued that individuals with ASD have a diminished capacity for “identifying” with the attitudes of others (a form of self-other relatedness) and this results in diminished awareness of self and others and, consequently, diminished communication and reciprocal social interaction (key diagnostic features). Frith (2003) has put forward a similar, if more extreme argument, proposing that people with autism have a “missing self” and, consequently, cannot “communicate with other people’s self-aware selves” (p. 216). She further proposes that the self is responsible for top-down control of cognition and behavior (i.e., self-regulation) and suggests that, ultimately, characteristic cognitive-level difficulties in theory of mind, executive functions, and central coherence (the capacity for global rather than local perceptual or cognitive processing) can be explained by this putative absent self.

As well as potentially explaining a large portion of the overall ASD phenotype, atypical self-awareness has been proposed as a key contributor to the specific profile of strengths and difficulties with memory experienced by individuals with this disorder (see Lind, 2010; Lind, Williams, Grainger, & Landsiedel, 2018). Although some have suggested that difficulties with self-awareness in ASD are limited to objective, second-order self-representation (the self as the *object* of experience—the “me”; e.g., Frith, 2012; Williams, Nicholson, & Grainger, 2018), others have suggested that subjective, first-order self-representation is also atypical in this disorder (the self as the subject of experience—the “I”; e.g., Powell & Jordan, 1996; Millward, Powell, Messer, & Jordan, 2000).

The performance of individuals with autism on self-referential memory tasks has been cited by many researchers as providing key insight into self-awareness in this disorder (e.g., Lombardo & Baron-Cohen, 2010). On the one hand, if the self-reference effect is diminished, this may be taken as evidence for impaired self-awareness. On the other hand, if people with ASD show a typical or enhanced self-reference effect, this may be taken as indirect evidence for intact or superior self-awareness in autism. Three studies (Burrows, Usher, Mundy, & Henderson, 2017; Henderson et al., 2009; Lombardo, Barnes, Wheelwright, & Baron-Cohen, 2007)^1^ have explored self-referential memory among people with ASD using the explicit trait memory paradigm. Across the studies, the mean weighted effect size for the between-groups difference in the size of the self-reference effect is Cohen’s *d* = 0.65, suggesting a moderate-to-large diminution of this effect in ASD.^2^ These results have frequently been interpreted as evidence of an impairment in objective self-awareness in this disorder. For example, some of the authors of the current paper have previously argued (Williams et al., 2018; Lind, 2010) that the self-reference effect is diminished in ASD because the classic trait memory paradigm requires explicit evaluative judgments about self (i.e., the self to be thought about) during the encoding phase of the task. As such, a diminished second-order self-representation among people with ASD means that self-referential information is not processed more deeply/preferentially than other-referential information, at *this* level. However, Williams et al. predicted that the self-bias would be undiminished among autistic people on an incidental encoding version of the trait memory paradigm, because encoding of self-relevant information required only subjective, first-order orientation to self-relevant information (the “I”), rather than explicit reflection on and evaluation of oneself (the “me”; see Cunningham et al., 2014, for the same argument about the underlying basis of performance on the implicit and explicit trait memory paradigms).

We tested these hypotheses in three experiments using the classic, explicit trait memory paradigm, as well as an implicit, incidental version of the task employed by Turk et al. (2008). In Experiment 1, we took an individual differences approach and investigated whether the size of the self-bias in each condition would be associated significantly with the number of self-reported ASD traits among a large sample of neurotypical (NT) adults. It is well established that number of ASD traits varies continuously in the general population, with the overwhelming majority of NT individuals manifesting at least some ASD traits and diagnosed individuals falling toward the upper end of the distribution (e.g., Baron-Cohen, Wheelwright, Hill, Raste, & Plumb, 2001; Constantino & Todd, 2003). The term “broad autism phenotype” describes individuals with elevated but subclinical levels of ASD traits (Bailey et al., 1995). The continuously distributed nature of ASD traits means that it is possible to explore the relation between these traits and other variables among people without an ASD diagnosis. This is the approach we took in Experiment 1. At the outset of the study, we predicted that the number of self-reported ASD traits would be significantly negatively associated with the size of the self-bias on the traditional explicit paradigm (more ASD traits = smaller self-bias), but not with the size of the self-bias on the incidental implicit paradigm.

## Experiment 1

### Method

#### Participants

Two-hundred-and-fifty-seven psychology students (198 female, 59 male) from the University of Kent or City, University of London took part in Experiment 1. This sample size allows detection of correlations of ≥.17 on 80% of occasions if they exist (G*Power3; Faul, Erdfelder, Buchner, & Lang, 2009). The average age of participants was 20.58 (*SD* = 5.37) years. No participant had a history of ASD, according to self-report. All participants also completed the autism-spectrum quotient (AQ), a valid and reliable measure of ASD traits in people (of normal intelligence) with a full diagnosis and in the general population (Baron-Cohen, Wheelwright, Skinner, Martin, & Clubley, 2001). Participants read statements (e.g., “I find social situations easy”; “I find myself drawn more strongly to people than to things”) and decide the extent to which each statement applies to them, responding on a 4-point Likert scale, ranging from 1 (definitely agree) to 4 (definitely disagree). Scores range from 0 to 50, with higher scores indicating more ASD traits. The mean AQ score for Experiment 1 participants was 15.49 (*SD* = 6.28; range = 3–34).

All participants gave informed consent and received course credit in partial fulfillment of their degree, for taking part in the study. The experiment was approved by the University of Kent (approval code: 201715096156554671) and City, University of London’s (approval code: PSYETH (U/L) 17/18 11) Psychology Research Ethics Committees.

#### Procedure and materials

Participants completed implicit and explicit encoding conditions of the trait memory task, always completing the implicit condition first. The stimuli comprised 144 trait words (half of which were psychological traits, such as “intelligent”, and half of which were physical traits, such as “tall”). All items were rated on a 5-point scale for valence by 10 independent adults. The mean ratings across raters were used to split the items into six equal lists of 12 traits (50% psychological, 50% physical), which were balanced for valence. A one-way analysis of variance (ANOVA) show no effect of list or trait type (or interaction between them) on valence, *F*s ≤ 0.02, *p*s ≥ .90. These individual lists were then organized into 12 different combinations to produce 12 different versions of the experiment, ensuring that each list of psychological and physical words was present within each of the six possible referent/encoding conditions (self-implicit, other-implicit, lure-implicit, self-explicit, other-explicit, lure-explicit). These 12 versions were used so that stimuli varied in relation to condition across participants.

Before beginning the tasks, participants were shown a picture of a famous person of the same gender and asked to identify them. Correctly identifying their name selected that name as the “other name” to be used during both the trait memory tasks. If the participant incorrectly identified the first person, they were shown a second famous person to identify. Females were first shown a picture of Queen Elizabeth, followed by Theresa May. Males were shown a picture of Prince Charles, followed by David Cameron. For each participant, the experimenter checked that the first name on the consent form was their preferred name. If it was not, the verbally stated preferred name was used. Participants were informed that throughout the experiment they would be seeing either their own name or the name of the correctly identified famous person, along with some other words and would have some simple responses to make in relation to the stimuli.

##### Implicit task

###### Encoding phase

Each trial began with a name (participant’s or celebrity’s) presented centrally on a computer monitor for 500ms, followed by the presentation of a trait word either to the left or right of the name for 1500ms, with the name remaining on screen throughout. The words then disappeared, and the participant was asked to press the “Z” or “M” key to indicate whether the trait word had appeared to the left or right, respectively, of the central name. Participants were instructed that they did not need to pay attention to either the name or the meaning of the trait word on each trial. Rather, their task was merely to concentrate on the spatial location of the trait word on each trial. Each participant performed 48 trials (fixed pseudorandomized order), 24 with their own name and 24 with the name of the celebrity.

###### Recognition test phase

Following completion of the encoding/study phase, participants completed the surprise recognition test phase. On each trial, participants were presented with a trait word along with the question, “Did you see this word during the study phase?” and a “yes” box and a “no” box. Participants had unlimited time to click in either response box to indicate whether or not they recognized the word from the study phase. Each participant performed 72 trials (fixed pseudorandomized order), 48 of which included a previously studied/old trait presented during the study phase and 24 of which included a previously unseen lure/new item. The fixed pseudorandomized order ensured that no more than 3 items from any category (self, other, lure) appeared in a row.

##### Explicit task

###### Encoding phase

Each trial began with a name (participant’s or celebrity’s) presented centrally for 500 ms, followed by the presentation of a trait word directly below the name for 1,500 ms. The words then disappeared, and the participants were instructed to press the “Z” key if the trait word was applicable to the named person (themselves/celebrity) or the “M” key if it did not apply.

Participants were reminded that the task was not a personality test and no-one would be judging their responses so they should respond as honestly as possible and make their best guess if they were unsure whether a trait word applied or not. Each participant performed 48 trials (fixed pseudorandomized order), 24 with their own name, and 24 with the name of the identified famous person.

###### Recognition test phase

The procedure for the recognition test phase in the explicit condition was identical to the procedure used in the implicit condition.

#### Variables and scoring

d′ (d-prime) scores were calculated as a standard measure of recognition memory accuracy, using the formula, *d*′ = *z*(HR) – *z*(FAR). Here, *HR* refers to hit rate (proportion of old item correctly identified as old); *FA* refers to false alarm rate (proportion of new items incorrectly identified as old); and *z* refers to *z* transformation. Higher *d*′ scores indicate greater discrimination between “old” and “new” items, and hence greater recognition memory accuracy. As a measure of the size of the self-reference effect, self-bias scores (self-other difference scores) were calculated by subtracting average other/celebrity trial *d*′ scores from average self trial *d*′ scores.

#### Data analysis

An increasingly used supplement to null hypothesis significance testing in general is to calculate a Bayes factor for each key analysis. Bayesian analyses provide an estimation of the relative strength of a finding for one hypothesis over another (i.e., the alternative hypothesis over the null, or vice versa), which allows a more graded interpretation of the data than is possible using *p* values or effect sizes alone (e.g., Dienes, 2014; Rouder, Speckman, Sun, Morey, & Iverson, 2009). According to Jeffreys’ (1961) criteria, Bayes factors (BF_10_) > 3 provide firm evidence for the alternative hypothesis (with values >10, >30, and >100 providing strong, very strong, and decisive evidence, respectively) and values under 1 provide evidence for the null (with values <0.33 providing firm evidence). BF_10_ values can be considered to reflect the likelihood that the alternative hypothesis is more likely to be true than the null hypothesis. Hence, a BF_10_ of 3 suggests the alternative hypothesis is three times more likely to be true than the null hypothesis. Bayesian analyses were conducted using JASP 0.8.1 (JASP Team, 2016).

### Results

#### Analyses of variance

In the implicit condition, the mean *d*′ was 1.49 (*SD* = 0.76) on self-referential trials and 1.31 (*SD* = 0.64) on other-referential trials (hit rates and false alarm rates are presented in Supplementary Table S1 in the online supplemental materials). In the explicit condition, the mean *d*′ score was 2.56 (*SD* = 0.84) on self-referential trials and 2.13 (*SD* = 0.80) on other-referential trials. A 2 (condition: implicit/explicit) × 2 (referent: self/other) repeated-measures ANOVA was conducted on this data. The main effects of referent, *F*(1, 256) = 178.62, *p* < .001, η_*p*_^2^ ≥ .41, and condition, *F*(1, 256) = 265.55, *p* < .001, η_*p*_^2^ ≥ .51, were significant, as was the interaction between them, *F*(1, 256) = 28.71, *p* = .001, η_*p*_^2^ = .10.

**Figure 1:**
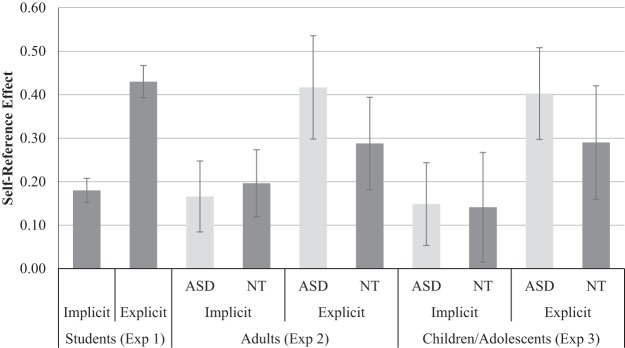
Mean self-reference effects (i.e., self-bias scores: self *d*′ minus other *d*′) from the implicit and explicit conditions of Experiments 1, 2, and 3. Error bars represent standard errors. ASD = autism spectrum disorder group; NT = neurotypical group.

Breaking down the interaction effect, planned contrasts indicated a significant self-reference effect (self *d*′ significantly greater than other *d*′) in both the explicit condition, *t*(256) = 11.78, *p* < .001, *d* = 0.74, BF^10^ > 100, and implicit condition, *t*(256) = 6.40, *p* < .001, *d* = 0.40, BF^10^ > 100. However, as shown in Figure 1, self-bias score was significantly larger in the explicit condition (*M* = 0.43, *SD* = .59; range = −1.49–2.88) than in the implicit condition (*M* = 0.18, *SD* = 0.45; range = −1.73–1.63), *t*(256) = 5.36, *p* < .001, *d* = 0.33, BF^10^ > 100.^3^

#### Association analyses

Kendall’s tau correlations were carried out to explore the relationships among AQ scores, implicit self-bias scores (self *d*′ minus other *d*′) and explicit self-bias scores. The correlation matrix is reported in Table 1 and accompanying scatterplots can be found in Supplementary Figure S1 in the online supplemental materials. All correlations were small and not statistically significant, with Bayes factors indicating firm support for the null hypothesis.[Table-anchor tbl1]

### Discussion

As expected, NT individuals showed a typical self-reference effect (self-bias) in memory, recognizing trait words processed in relation to self significantly more reliably than trait words processed in relation to others in both the implicit and explicit conditions of the trait memory task. In keeping with our a priori prediction, AQ score was not significantly associated with the size of the self-bias in the implicit condition. Contrary to our prediction, however, AQ score was not significantly associated with the size of the self-bias in the explicit condition either. There was sufficient statistical power to detect even small associations if they existed, and Bayesian analyses consistently suggested that the data provided firm support for the null hypotheses in association analyses. Of course, the majority of the sample was female and the participants tested had a relatively narrow age range. A sample more representative of the general population would have been advantageous and increased confidence in the generalizability of results. However, post hoc analyses (see Footnote 3) showed that sex/gender did not influence the size of the self-bias or size of the correlation with AQ in Experiment 1 and so it is not clear that a sample with a higher proportion of males would have changed the results.

The current results are out of keeping with those of Henderson et al. (2009) who observed a significant association between number of ASD traits (measured using the Autism Spectrum Screening Questionnaire; ASSQ; Ehlers, Gillberg, & Wing, 1999) and the size of the self-bias on an explicit trait memory paradigm that was very similar to the explicit condition used in the current study. Of course, Henderson et al.’s study used a case-control design and observed a significant association between ASSQ score and size of self-bias (after controlling for group differences in the size of the self-bias) among their 59 participants (note that groups were collapsed on the basis that a Group × Self-Bias interaction effect emerged in their regression analysis, although the statistics associated with the interaction effect were not reported).

Given that ASD features are likely to be distributed continuously throughout the general population (e.g., Frazier et al., 2014), studying individual differences in ASD traits and their relation to psychological abilities in the NT population can make an important contribution to our understanding of ASD itself. However, there can still be qualitative differences in the mechanisms/processes that underpin those traits in each population (e.g., Mandy et al., 2012; Peterson, Wellman, & Liu, 2005). As such, a full understanding requires the study of diagnosed cases, as well as traits in the NT population. Thus, even though we found no evidence of an association between ASD traits and size of the self-bias in individuals from the general population in Experiment 1, it does not rule out the possibility that between-groups differences in the size of the self-bias (and/or significant AQ × size of self-bias associations) would emerge in a case-control study. Therefore, in Experiment 2, we gave the same implicit and explicit conditions of the trait memory task used in Experiment 1 to a group of adults with a full diagnosis of ASD, as well as a closely matched group of NT comparison adults. At the outset of the study, we predicted that the size of the self-bias would be significantly smaller among ASD participants than comparison participants in the explicit condition only.

## Experiment 2

### Method

#### Participants

Thirty autistic adults (eight women and 22 men) and 30 NT adults (six women and 24 men) took part in Experiment 2. The number of women and men did not differ significantly between groups, χ^2^(1, *N* = 60) = 0.37, *p* = .761, φ = .08. Participants in the ASD group had received verified diagnoses, according to conventional criteria (American Psychiatric Association, 2000; World Health Organization, 1993) and 29/30 agreed to complete the Autism Diagnostic Observation Schedule (ADOS; Lord et al., 2000), a detailed observational assessment of ASD features (with sensitivity of 80.4–100.0% and specificity of 18.2–73.6%; Risi et al., 2006), which was administered by a research-reliable assessor. All participants completed the AQ (Baron-Cohen et al., 2001), which has sensitivity of .95 and specificity of .52 (Woodbury-Smith, Robinson, Wheelwright, & Baron-Cohen, 2005). ADOS and AQ scores were obtained as descriptive measures to characterize the samples and for the purpose of association analyses, rather than as inclusion/exclusion criteria (given that neither are intended as stand-alone diagnostic tools and that neither has perfect sensitivity or specificity; see Bishop, 2011, for relevant arguments).

ADOS social + communication scores ranged from 0 to 21. One participant with ASD did not wish to complete the ADOS and six scored below the social + communication cut-off of ≥7 points (with an ASD sample of *n* = 30 and sensitivity of 80.4–100%, we should expect to find 0–6 false negatives), but each of these individuals met or exceeded the recommended (Woodbury-Smith et al., 2005) AQ cut-off of ≥26 points (range: 32–41). Participants with ASD scored between 18 and 47 on the AQ, with five scoring below the AQ cut-off (with an ASD sample of *n* = 30 and sensitivity of 95% we should expect to find 1–2 false negatives), but all of those individuals scored ≥9 points on the ADOS (range = 9–21). Hence, all participants in the ASD group had a verified clinical diagnosis and all scored above the cut-off on either the ADOS or the AQ. All but one NT participant scored <26 on the AQ (scoring 29; with a NT sample of *n* = 30 and specificity of 52% we should expect to find 15–16 false negatives). Results were identical after excluding participants with ASD who scored <7 on the ADOS (or had missing data) or <26 on the AQ and NT participants who scored ≥26 on the AQ (see Part 2 of the online supplemental materials).

All participants also completed the Wechsler Abbreviated Scale for Intelligence-II (Wechsler, 1999), which provides verbal, performance, and full-scale IQ scores. We also included two widely used measures of mindreading (Reading the Mind in the Eyes; Baron-Cohen et al., 2001; and Animations; Abell, Happé, & Frith, 2000) as a “control” to ensure that our ASD group was reasonably representative of the wider ASD population in showing difficulties in this area (details of the methods are included in Part 2A of the online supplemental materials). Participant characteristics and group matching statistics are presented in Table 2. No participant in either group reported current use of psychotropic medication or illegal recreational drugs, and none reported any history of neurological or psychiatric illness other than ASD. All participants gave informed consent and received £7.50 per hour for their time plus travel expenses. The current study received ethical approval from the University of Kent Psychology Research Ethics Committee (approval code: 201715096156554671).[Table-anchor tbl2]

#### Procedure and materials

Participants from each group completed each condition (implicit/explicit) of the trait memory paradigm used in Experiment 1.

#### Power analysis

At the outset of the study, we calculated the average weighted effect size (*d*) for the between-groups (ASD/comparison) difference in the size of the self-bias on the traditional (explicit) trait memory paradigm across the studies by Lombardo et al. (2007); Henderson et al. (2009), and Burrows et al. (2017). (See Footnote 2). The resulting *d* value was 0.65. An *a priori* power calculation using G*Power3 (Faul et al., 2009) revealed that to detect a between-groups difference of this magnitude on 80% of occasions, 60 participants (30 per group) were required. Thus, the current study was powered to detect the predicted difference if it existed.

### Results

#### Analyses of variance

Table 3 shows descriptive statistics for *d*′ scores in each condition and for each variable of the implicit and explicit tasks. A 2 (condition: implicit/explicit) × 2 (referent: self/other) × 2 (group: ASD/NT) mixed ANOVA was conducted on this data. Results are reported in Table 4 and illustrated in Figure 1. Both main effects of referent and condition were significant, and the interaction between them was near to statistical significance (*p* = .08). This nearly significant interaction was driven by the same pattern of results as observed in Experiment 1 (significant self-reference effect in both conditions, but larger in the explicit than implicit condition).[Table-anchor tbl3][Table-anchor tbl4]

Crucially, none of the effects involving group even approached significance. To be clear, there was no significant between-groups difference in the self-bias score (self *d*′ minus other *d*′) in either the explicit condition, *t*(58) = 0.81, *p* = .422, *d* = 0.21, BF^10^ = 0.35, or implicit condition, *t*(58) = 0.27, *p* = .787, *d* = 0.07, BF^10^ = 0.27 (descriptive statistics for self-bias scores are presented in Table 3). The self-reference effect (difference between self *d*′ and other *d*′) in the explicit condition was significant among both participants with ASD, *t*(29) = 3.51, *p* = .002, *d* = 0.64, BF^10^ = 23.18, and NT participants, *t*(29) = 2.71, *p* = .011, *d* = 0.50, BF^10^ = 4.09. Likewise, the self-reference effect in the implicit condition was significant among both participants with ASD, *t*(29) = 2.03, *p* = .026 (one-tailed), *d* = 0.37, BF^10^ = 1.17, and NT participants, *t*(29) = 2.55, *p* = .016, *d* = 0.46, BF^10^ = 2.98.

#### Association analyses

Kendall’s tau correlations were carried out to explore the relationships among AQ scores, implicit self-bias scores (self *d*′ minus other *d*′) and explicit self-bias scores within the ASD group, the NT group, and the total, combined sample. The correlation matrix is reported in Table 1. All correlations were small and not statistically significant, with Bayes factors indicating support or firm support for the null hypothesis.

Additional correlations were carried out to explore the relation between ADOS social + communication score and self-bias scores within the ASD group (only this group completed the ADOS). Consistent with the findings above, ADOS social + communication score was not significantly associated with self-bias score in either the implicit condition, *r*_τ_ = .05, *p* = .710, BF^10^ = 0.26, or explicit condition *r*_τ_ = .13, *p* = .35, BF^10^ = 0.37. It is worth noting that seven out of the eight (small and nonsignificant) correlations we ran between AQ/ADOS and self-bias scores were positive (i.e., in the direction reflecting more ASD traits = larger self-bias).

### Discussion

We found no evidence that the size of the self-bias on either the traditional explicit trait memory paradigm or the new implicit/incidental paradigm was diminished among adults with ASD. Both groups showed a significant self-bias in both implicit and explicit conditions, and the between-groups difference in the size of these self-biases was small and nonsignificant in each case. Bayesian analyses consistently suggested that the data supported the null hypothesis with respect to group differences on the task. Furthermore, the number of ASD traits (measured with AQ or ADOS) was not significantly associated with the size of the self-bias in either the implicit or explicit condition among either ASD or NT participants.

At the outset of the study, we had predicted that the size of the self-bias in the explicit condition would be significantly diminished in participants with ASD. Our prediction was based on our initial reading of the literature, but the unexpected null findings from the current study (and from Experiment 1) led us to reengage with the relevant literature to consider possible causes of the discrepancy between results from our study and results from previous studies. Upon rereading the relevant papers, two things stood out to us, namely the age of samples and the closeness of group matching across studies.

With regard to the issue of age, it was notable that Lombardo et al. (2007) assessed the size of the self-bias on a traditional explicit trait memory paradigm among adults with ASD, whereas Henderson et al. and Burrows et al. explored this in children/adolescents with ASD. Whereas Henderson et al. and Burrows et al. reported that the size of the self-bias was significantly diminished in their participants with ASD, the group differences in Lombardo et al. were actually nonsignificant. In Lombardo et al.’s study, both ASD (*n* = 30) and comparison (*n* = 30) participants showed a significant self-bias both when the other person referent was a friend and (as in the current study) when the other person was a familiar famous person. The between-groups difference in the size of the self-bias found by Lombardo et al. was small and nonsignificant in both the friend contrast (*p* = .95, *d* = 0.02) and famous other contrast (although this latter difference approached significance; *p* = .07. *d* = 0.49). In contrast, the between-groups difference in the size of the self-bias was moderate-to-large and significant in both Henderson et al., (*d* = 0.66) and Burrows et al. (*d* = 0.75; see Footnote 2). One possibility that might explain contradictory findings in the literature is that the influence of self-reference (explicit and/or implicit) on memory is diminished in children with ASD, but not in adults with ASD. As Williams and Bowler (2014) noted, “we should never forget that the clinical picture we see among individuals with a diagnosis of ASD represents a particular point in an atypical developmental trajectory, in which both the clinical features and any putative underlying factors may be in a process of change” (p. 5).

It may be that early disruption of the link between self and memory resolves, or is compensated for, by the time autistic individuals reach adulthood. Therefore, in Experiment 3, we gave the implicit and explicit conditions of the trait memory task to a group of children/adolescents with a full diagnosis of ASD, as well as a closely matched group of NT comparison children/adolescents. The average age of participants was very similar to (and nonsignificantly different from) the average age of participants in the studies by Henderson et al. and Burrows et al. so that this “developmental hypothesis” could be tested.

## Experiment 3

### Method

#### Participants

Twenty-six autistic children/adolescents (7 girls and 19 boys) and 26 neurotypical children/adolescents (8 girls and 18 boys) took part in Study 3. The number of girls and boys did not differ significantly between groups, χ^2^(1, *N* = 52) = 0.09, *p* = .760, φ = .04. Participants in the ASD group had received verified diagnoses, according to conventional criteria (American Psychiatric Association, 2000; WHO, 1993). The parents of all completed the Social Responsiveness Scale (SRS; Constantino et al., 2003), which is a 65-item parent-report questionnaire assessing autism traits, with sensitivity and specificity of 85% and 75%, respectively (Constantino & Gruber, 2005). SRS *t*-scores < 60 are considered normal range; 60–75 = mild-moderate range; ≥ 75 = severe range. SRS scores were obtained as descriptive measures to characterize the samples and for the purpose of association analyses, rather than as inclusion/exclusion criteria (given that the SRS is not intended as a stand-alone diagnostic tool and does not have perfect sensitivity or specificity; see Bishop, 2011, for relevant arguments).

All but one participant with ASD scored over the cut-off of 60 on the SRS (with an ASD sample of *n* = 26 and a sensitivity of 85% we should expect three to four false negatives). Scores ranged between 55 and 90. All but two NT participants scored below 60 on the SRS (range 35–90), with those two participants scoring 75 and 90 (with a NT sample of *n* = 26 and a specificity of 75%, we should expect six to seven false positives). Results were identical after excluding and participants with ASD who scored <60 on the SRS and NT participants who scored ≥60 on the SRS (see Part 3 of the online supplemental materials).

All participants completed the Wechsler Abbreviated Scale for Intelligence-II (Wechsler, 1999), which provides verbal, performance, and full-scale IQ scores. We also included two widely used measures of mindreading (child version of Reading the Mind in the Eyes; Baron-Cohen et al., 2001; and Animations; Abell et al., 2000) as a “control” to ensure that our ASD group was reasonably representative of the wider ASD population in showing difficulties in this area (details of the methods are included in Part 3A of the online supplemental materials). Participant characteristics and group matching statistics are shown in Table 5. All participants and their parents gave informed consent. The study received ethical approval from the University of Kent Psychology Research Ethics Committee (approval code: 201715096156554671).[Table-anchor tbl5]

#### Power analysis

Experiments 1 and 2 were planned at the outset of the research program. Experiment 3 was conducted only after results from Experiments 1 and 2 were contrary to expectations and after we had rereviewed the relevant literature. At that stage, we calculated the average weighted effect size (Cohen’s *d*) for the between-groups (ASD/comparison) difference in the size of the self-bias only in the studies of children with ASD by Henderson et al. (2009) and Burrows et al. (2017). The resulting *d* value was 0.72. An *a priori* power calculation using G*Power3 (Faul et al., 2009) revealed that to detect a between-groups difference of this magnitude on 80% of occasions, 50 participants (25 per group) were required. Thus, the current study was adequately powered to detect the predicted difference if it existed. If working on the assumptions we had when beginning Experiment 2 that the average weighted effect size was 0.65, rather than 0.72, then power was .75 in Experiment 3.

#### Procedure and materials

Participants from each group completed the implicit and explicit conditions of the trait memory task. Given that some of the words used in Experiments 1 and 2 might be too advanced for some children/adolescents to comprehend, a different set of stimuli was used. These stimuli were designed to be equivalent (in terms of mean written word frequency, number of syllables, and valence) to those used by Henderson et al. (2009) in their study of the self-reference effect (using the trait memory paradigm) among children/adolescents with ASD. One-hundred-and-two psychological trait words (21 of which were used in Henderson et al.’s study) were divided into six lists of 17 words, and these lists were then combined into 6 different versions. Within each version, each list represented a different one of the six possible conditions (self-implicit, other-implicit, lure-implicit, self-explicit, other-explicit, and lure-explicit). This ensured that words lists were counterbalanced across participants in relation to the condition they reflected. The six lists contained equal numbers of positively and negatively valanced words and a multivariate analysis of variance indicated that they were equated for mean number of syllables and KF written word frequency, *F*(10, 156) < .01, *p* > .99, η_*p*_^2^ <.01.

During the encoding phases of both the implicit and explicit conditions, each participant undertook 34 trials (fixed pseudorandomized order)—17 with their own name and 17 with the name of the identified famous person’s name. During the recognition test phases of both the implicit and explicit conditions, each participant performed 51 trials (34 trials from encoding phase, plus 17 previously unseen lure words). Because of the age of the participants in Experiment 3, we replaced the famous pictures used in Experiments 1 and 2 with pictures of famous people more likely to be identified by younger people. For the female participants, we used Emma Watson, Ariana Grande, Kim Kardashian, and Taylor Swift. For the male participants, we used Ed Sheeran, Justin Bieber, Dan TDM, and Pewdiepie. We used a pool of four famous males and females in Experiment 3 given the possibility that younger participants might not correctly identify some of the pictures. All other procedural elements were identical to those used in Experiments 1 and 2.

### Results

#### Analyses of variance

Table 6 shows descriptive statistics for *d*′ scores in each condition and for each variable of the implicit and explicit tasks. A 2 (condition: implicit/explicit) × 2 (referent: self/other) × 2 (group: ASD/NT) mixed ANOVA was conducted on this data. Results are reported in Table 7 and illustrated in Figure 1. Both main effects of referent and condition were significant, and the interaction between them was near to statistical significance (*p* = .09). This nearly significant interaction was of the same magnitude as observed in both Experiments 1 and 2, and reflected the same pattern of results (significant self-bias in both conditions, but larger in the explicit than implicit condition).[Table-anchor tbl6][Table-anchor tbl7]

Crucially, none of the ANOVA effects involving group even approached significance. There was no significant between-groups difference in self-bias score in either the explicit condition, *t*(50) = 0.67, *p* = .505, *d* = 0.19, BF^10^ = 0.34, or implicit condition, *t*(50) = 0.05, *p* = .964, *d* = 0.01, BF^10^ = 0.28. The self-reference effect (i.e., the difference between self *d*′ and other *d*′) in the explicit condition was significant among both participants with ASD, *t*(25) = 3.82, *p* < .001, *d* = 0.75, BF^10^ = 42.08, and NT participants, *t*(25) = 2.22, *p* = .036, *d* = 0.44, BF^10^ = 3.23. However, when the size of the self-bias in the implicit condition was analyzed in each group separately, it was nonsignificant (even when reported one-tailed) in either participants with ASD, *t*(25) = 1.56, *p* = .066, *d* = 0.31, BF^10^ = 1.12, or NT participants, *t*(25) = 1.12, *p* = .273, *d* = 0.22, BF^10^ = 0.62.^4^

#### Association analyses

Kendall’s tau correlations were carried out to explore the relationships among SRS scores, implicit self-bias scores (self *d*′ minus other *d*′) and explicit self-bias scores within the ASD group, the NT group, and the total, combined sample. The correlation matrix is reported in Table 1. All correlations were small and nonsignificant, with Bayes factors indicating support or firm support for the null hypothesis. Five out of the six (small and nonsignificant) correlations between SRS and self-bias scores were positive (i.e., in the direction reflecting more ASD traits = larger self-bias).

### Discussion

We found no evidence that the size of the self-bias on either the traditional explicit trait memory paradigm or the new implicit/incidental paradigm was diminished among children with ASD. Participants showed a self-bias in both implicit and explicit conditions, and the between-groups difference in the size of these self-biases was small and nonsignificant in each case. Bayesian analyses consistently suggested that the data supported the null hypothesis with respect to group differences on the task. Furthermore, the number of ASD traits reported was not significantly associated with the size of the self-bias in either the implicit or explicit condition among either ASD or NT participants.

## General Discussion

In keeping with predictions, the size of the self-bias in the implicit condition was not significantly associated with the number of ASD traits manifested in any of five participant groups across the three experiments, and there was no significant between-groups difference in the size of the self-bias in either of the case-control Experiments 2 or 3. These important findings are the first of their kind, to our knowledge, and suggest that both adults and children/adolescents with ASD implicitly process self-relevant information in a preferential manner to at least the same extent as NT adults and children/adolescents do. These findings fit well with previous findings that the size of the self-bias on action memory tasks (i.e., enactment effect; see Grainger, Williams, & Lind, 2016), as well as the size of self-bias following speeded perceptual judgments on a shapes task (Williams et al., 2018), are undiminished among people with ASD. Together, they imply that self-experience (the “I”) influences cognition and perception in a typical manner among people with ASD, which suggests in turn that self-experience itself is typical in this disorder (contrary to the suggestions of some including Powell & Jordan, 1996, and Russell, 1996). Thus, the current findings represent a highly novel contribution to the literature. Arguably, however, other aspects of the results are equally as important.

Contrary to predictions, however, we found no evidence that self-reference (a) influenced memory to a lesser degree among autistic individuals than NT individuals (Experiments 2 and 3) or (b) was related to number of autistic traits (Experiments 1, 2 and 3) in the explicit condition of the task. It is important to note that our previous theoretical contributions have included the assumption that objective self-awareness is diminished in ASD and that self-reference effects on tasks requiring such self-awareness are diminished in ASD (see Grisdale, Lind, Eacott, & Williams, 2014; Lind, 2010; Williams, 2010). We deliberately powered our studies to detect the predicted (moderately sized) between-groups differences in the size of the explicit self-bias (Experiments 2 and 3), and to detect the predicted (moderately sized) association between the size of the explicit self-bias and number of self-reported ASD traits (Experiment 1). Moreover, we used Bayesian analyses to provide support for the null hypothesis that we predicted with respect to the results from the implicit condition of the task (i.e., lack of between-groups differences in the size of the implicit self-bias and lack of a reliable association with number of ASD traits). The fact that Bayesian analyses consistently suggested the diagnostic groups in each of Experiments 2 and 3 showed a reliable explicit self-bias and were also equivalent with respect to the size of the self-bias, strongly suggests that our *a priori* hypotheses were not supported by the data. Indeed, it is striking that participants with ASD in both experiments had a numerically (if not statistically significantly) larger explicit self-bias than did NT comparison participants. Moreover, the association between number of ASD traits and size of the explicit self-bias was positive rather than negative in every analysis (average *r*_τ_ = .15).

How should we explain the discrepancy between the current set of results and those results from other studies that have used the trait memory paradigm among individuals with ASD? On reflection, our results from Experiment 2 were not out of keeping with those from the only other study of adults with ASD to explore the self-bias on the explicit trait memory paradigm; in fact, Lombardo et al. (2007) did not find significant between-groups differences in the size of the self-bias either when the other person evaluated in the encoding condition was a friend or a famous other. Therefore, the only two relevant studies among autistic adults (the current study and Lombardo et al.’s) have each failed to find a significant diminution of the self-bias on the explicit paradigm. The results from previous studies of the explicit self-bias in children with ASD are arguably more difficult to interpret. In Henderson et al.’s study, results showed clearly that the self-bias was diminished among children/adolescents with this disorder. Burrows et al.’s (2017) data also appeared to show this, but it should be noted that the groups in Burrows et al.’s study did not appear to be matched for age, VIQ, PIQ, or sex ratio. Burrows et al. attempted to overcome this possible confound by covarying age, VIQ, and sex (but not PIQ) in a series of analyses of covariance (ANCOVAs). Unfortunately, this approach does not overcome the problem of unmatched groups and ANCOVA should not be used when groups are not matched on the covariates (see Miller & Chapman, 2001; also Williams, Peng, & Wallace, 2016). It is impossible to know whether the failure to match groups in their study contributed to the finding of between-groups differences in the size of the self-bias. Either way, our aim is not to criticize the study by Burrows et al. but merely to consider possible reasons for the discrepancy between their results and ours.

Although the source of the discrepancy in results across studies is not entirely clear, one take-home message might be that researchers should be cautious about drawing absolute conclusions on the basis of results from a limited pool of studies. Despite our theoretical inclinations, it may be that explicit self-reference effects are typical and undiminished among people with ASD. The fact that two studies from the same laboratory have observed a diminished explicit self-bias in adolescents with ASD (Burrows et al., 2017; Henderson et al., 2009) should not lead researchers to view the case as closed, so-to-speak, with regard to self-reference effects in ASD. Of course, null findings are often scrutinized particularly heavily and it is important to rule out potential confounds that may have led to the null results. We have two points to make in this regard. First, the current findings are not null, in general; both participants with and without ASD showed significant self-biases (the effects were present and thus not null). Rather, the results were null specifically with respect to between-groups differences in the size of these self-biases. Our ASD groups were representative in scoring in the ASD range on measures of feature severity and in showing the classic mindreading impairments that are known to affect people with this disorder, so it is unclear how confounds in our experiments could have artificially produced significant self-biases. Second, it is important to note that science (and perhaps psychology, in particular; see Fanelli, 2010) is subject a series of biases that unduly favor publication of results that support alternative hypotheses. Through biases of selection (i.e., the “file drawer problem”; Rosenthal, 1979) and inflation (i.e., selective reporting/p-hacking; e.g., Masicampo & Lalande, 2012; Kühberger, Fritz, & Scherndl, 2014), our understanding of psychological phenomena is almost certain to be detrimentally affected. Therefore, it is particularly important to publish null results if we are to avoid biasing the field unduly. In the current study, we expected to find between-groups differences in the size of the explicit self-bias and powered our experiments accordingly. The fact that we did not find such a diminution in either of two experiments should lead to a high degree of caution when drawing the conclusion that explicit (or implicit) self-reference effects are diminished in people with ASD.

If the current results are valid and reliable (the degree of consistency across the three experiments, even though the samples differed considerably in terms variables such as gender ratio and age, suggests they probably are), they suggest that subjective and objective levels of self-awareness are intact in ASD, or at least sufficiently intact to bias attention and memory in a typical manner. Of course, the results do not show that all types of self-related information are represented among people with ASD. Indeed, there is substantial evidence for impairments in awareness of own mental states (metacognition) and this comes from a variety of studies using several different kinds of task (e.g., Cooper, Plaisted-Grant, Baron-Cohen, & Simons, 2016; Grainger, Williams, & Lind, 2014, 2016; Nicholson, Williams, Grainger, Lind, & Carruthers, 2019; Williams, Bergström, & Grainger, 2016). Thus, a difficulty with *meta-representing* oneself may be a specific problem with self-awareness in ASD (see Williams, 2010). Moreover, the results of Experiments 2 and 3 may have been different if intellectually low-functioning (IQs <70), rather than high-functioning, adults and children had been included. Perhaps intellectually low-functioning individuals have a more pervasive limitation with self-awareness that would manifest as a diminished self-bias on the type of experimental task used in the current study. Such a result would be theoretically important and clinically relevant, but we have no specific reason to believe this is the case at this time. Indeed, any such study with intellectually low-functioning individuals with ASD would need to include a carefully matched group of control participants with intellectual impairment to show that a diminished self-bias was associated with (or caused by) ASD specifically rather than co-occurring intellectual difficulties.

It may also be fruitful to use the self-referential memory paradigm for other purposes and to address different questions in future research. For example, it might be interesting to include objective/independent measures of each participant’s traits (e.g., by gathering reports from a close relative or friend). These independent trait ratings could then be compared to the trait ratings (i.e., patterns of trait endorsement) made by the participant during the encoding phase of the explicit trait memory paradigm. Theoretically, the closer the correspondence between the participant’s subjective reports of their traits and independent ratings of their traits, the better the participant’s self-awareness/meta-awareness of their personality. Perhaps the degree of metaknowledge would predict the size of the self-reference effect in recognition memory, at least among NT individuals. This is pure speculation, of course, but might indicate a new way to understand self-referential encoding processes among NT and autistic people.

In sum, the experiments presented here cast significant doubt on the common assumption (and one that we ourselves have held until now) that people with ASD have diminished (or atypical) objective self-awareness and blanket difficulties with self-referential cognition. We have a nagging suspicion that this area of research may have fallen prey to a misleading publication bias. So, as a final thought, we would like to encourage researchers in the field, who may have unpublished data from experiments using robust methods showing undiminished (or, equally, enhanced or diminished) performance by people with ASD on “self tasks” sitting in their proverbial “file drawers”; (Rosenthal, 1979), to try to disseminate those data. Perhaps then, we will gain a fuller understanding of these important issues.

## Supplementary Material

10.1037/abn0000467.supp

## Figures and Tables

**Table 1 tbl1:** Correlation Matrix (Kendall’s Tau) Showing the Relation Between Autism Traits, Implicit Self-Bias Memory Scores, and Explicit Self-Bias Memory Scores in Experiments 1, 2, and 3

Variable	1	2	3
1. Autism traits			
Experiment 1 (adult students)		−.05^a^	.06^a^
NT (*N* = 257)			
Experiment 2 (adults)			
ASD (*n* = 30)		.11^a^	.21^b^
NT (*n* = 30)		−.05^a^	.04^a^
Total (*N* = 60)		.01^a^	.12^b^
Experiment 3 (children/adolescents)			
ASD (*n* = 26)		.06^a^	.21^b^
NT (*n* = 26)		−.04^a^	.19^b^
Total (*N* = 52)		.05^a^	.17^b^
2. Implicit self-bias score			
Experiment 1 (adult students)	−.05^a^		−.02^a^
NT (*N* = 257)			
Experiment 2 (adults)			
ASD (*n* = 30)	.11^a^		.11^b^
NT (*n* = 30)	−.05^a^		−.02^a^
Total (*N* = 60)	.01^a^		.03^a^
Experiment 3 (children/adolescents)			
ASD (*n* = 26)	.06^a^		−.08^a^
NT (*n* = 26)	−.04^a^		.03^a^
Total (*N* = 52)	.05^a^		<−.01^a^
3. Explicit self-bias score			
Experiment 1 (adult students)			
NT (*N* = 257)	.06^a^	−.02^a^	
Experiment 2 (adults)			
ASD (*n* = 30)	.21^b^	.11^b^	
NT (*n* = 30)	.04^a^	−.02^a^	
Total (*N* = 60)	.12^b^	.03^a^	
Experiment 3 (children/adolescents)			
ASD (*n* = 26)	.21^b^	−.08^a^	
NT (*n* = 26)	.19^b^	.03^a^	
Total (*N* = 52)	.17^b^	<.01^a^	
*Note*. NT = neurotypical; ASD = autism spectrum disorder; BF = Bayes factor. Autism traits were measured with the Autism-spectrum Quotient in Experiments 1 and 2 and with the Social Responsiveness Scale in Experiment 3. All *p*s > .05.			
^a^ BF^10^ = .00–.33 (firm support for null hypothesis). ^b^ BF^10^ = .34–.99 (support for the null hypothesis).			

**Table 2 tbl2:** Experiment 2 Participant Characteristics and Group Matching Statistics

Variable	ASD (*n* = 30)	NT (*n* = 30)	*t*(58)	*p*	*d*
	*M* (*SD*)	*M* (*SD*)			
Age (years)	34.89 (11.32)	39.31 (13.97)	1.35	.184	.35
VIQ	103.33 (12.80)	105.70 (10.08)	.80	.430	.21
PIQ	102.93 (20.09)	105.60 (11.72)	.63	.533	.16
AQ	32.47 (7.52)	15.97 (5.49)	9.71	<.001	2.51
ADOS	10.10 (4.32)	—	—	—	—
RMIE	.66 (.18)	.78 (.10)	3.18	.002	.80
Animations	.58 (.30)	.72 (.22)	2.07	.040	.53
*Note*. ASD = autism spectrum disorder; NT = neurotypical; VIQ = verbal IQ; PIQ = performance IQ; FSIQ = full scale IQ; AQ = Autism-spectrum Quotient (total score; cut-off = 32); ADOS = Autism Diagnostic Observation Schedule (social + communication score; cut-off = 7); RMIE = Reading the Mind in the Eyes (proportion accuracy).					

**Table 3 tbl3:** Experiment 2 Descriptive Statistics for d′ (Recognition Memory Accuracy) Measures in Each Condition Among ASD and Neurotypical Participants

Condition and measure	ASD (*n* = 30)		NT (*n* = 30)		Total (*N* = 60)	
	*M* (*SD*)	Range	*M* (*SD*)	Range	*M* (*SD*)	Range
Implicit						
Self *d*′	1.32 (.64)	.16–2.88	1.17 (.73)	−.21–2.85	1.25 (.69)	−.21–2.88
Other *d*′	1.15 (.68)	.00–2.70	.97 (.68)	−.29–2.14	1.07 (.68)	−.29–2.70
Self-bias	.17 (.45)	1.06–1.49	.20 (.42)	−.78–1.41	.18 (.43)	−1.06–1.49
Explicit						
Self *d*′	2.33 (.70)	.92–3.46	2.32 (.74)	.97–3.77	2.32 (.71)	.92–3.77
Other *d*′	1.91 (.76)	.32–3.42	2.03 (.66)	1.11–3.46	1.97 (.66)	.32–3.46
Self-bias	.42 (.65)	−1.18–1.41	.29 (.58)	−.76–1.30	.35 (.61)	−1.18–1.41
*Note*. Self bias = self *d*′ minus other *d*′. ASD = autism spectrum disorder; NT = neurotypical.						

**Table 4 tbl4:** Analysis of Variance Results From Experiment 2 (Dependent Variable = d′ Score)

Variable	*F*(58)	*p*	η_*p*_^2^	Direction of effect
Condition	94.41	**<.001**	.62	Explicit > implicit
Referent	28.76	**<.001**	.33	Self > other
Group	.20	.658	.003	—
Condition × Referent	3.22	.078	.05	—
Condition × Group	1.21	.275	.02	—
Referent × Group	.24	.623	.004	—
Condition × Group × Referent	.70	.407	.01	—
*Note*. The bold type indicates statistically significant effects.				

**Table 5 tbl5:** Experiment 3 Participant Characteristics and Group Matching Statistics

Variable	ASD (*n* = 26)	NT (*n* = 26)	*t*(50)	*p*	*d*
	*M* (*SD*)	*M* (*SD*)			
Age (years)	12.64 (1.52)	13.28 (1.62)	1.48	.146	.41
VIQ	105.23 (10.18)	110.23 (11.83)	1.63	.109	.45
PIQ	108.23 (13.26)	114.12	13.92	.125	.43
SRS T-score	83.85 (9.67)	47.00 (11.74)	12.35	<.001	3.43
RMIE	.69 (.08)	.73 (.09)	1.53	.133	.43
Animations	.45 (.25)	.69 (.18)	4.13	<.001	1.15
*Note*. ASD = autism spectrum disorder; NT = neurotypical; VIQ = verbal IQ; PIQ = performance IQ; SRS = Social Responsiveness Scale T-Score (scores < 60 = normal range; 60–75 = mild-moderate ASD range; ≥ 75 = severe ASD range); RMIE = Reading the Mind in the Eyes (proportion accuracy).					

**Table 6 tbl6:** Experiment 3 Descriptive Statistics for d′ (Recognition Memory Accuracy) Measures in Each Condition Among ASD and Neurotypical Participants

Condition and measure	ASD (*n* = 26)		NT (*n* = 26)		Total (*N* = 52)	
	*M* (*SD*)	Range	*M* (*SD*)	Range	*M* (*SD*)	Range
Implicit						
Self *d*′	2.03 (.68)	.94–3.78	2.07 (.77)	.60–3.45	2.05 (.72)	.60–3.78
Other *d*′	1.88 (.64)	.62–3.13	1.93 (.82)	.00–3.08	1.91 (.73)	.00–3.13
Self-bias	.15 (.49)	−.84–1.11	.14 (.64)	−1.19–1.31	.14 (.56)	−1.19–1.31
Explicit						
Self *d*′	2.72 (.74)	1.19–3.78	2.72 (.74)	.50–3.78	2.71 (.74)	.50–3.78
Other *d*′	2.31 (.58)	.81–3.45	2.43 (.78)	.71–3.78	2.37 (.69)	.71–3.78
Self-bias	.40 (.54)	−.96–1.35	.29 (.67)	−.96–1.51	.35 (.60)	−.96–1.51
*Note*. ASD = autism spectrum disorder; NT = neurotypical. Self bias = self *d*′ minus other *d*′.						

**Table 7 tbl7:** Analysis of Variance Results From Experiment 3 (Dependent Variable = d′ Score)

Variable	*F*(50)	*p*	η_*p*_^2^	Direction of effect
Condition	24.36	**<.001**	.33	Explicit > implicit
Referent	18.11	**<.001**	.27	Self > other
Group	.16	.692	<.01	—
Condition × Referent	3.07	.086	.06	—
Condition × Group	<.01	.946	<.01	—
Referent × Group	.27	.606	.01	—
Condition × Group × Referent	.21	.648	<.01	—
*Note*. The bold type indicates statistically significant effects.				

## Footnotes

1 Toichi et al. (2002) also investigated self-reference effects in ASD but their study was not equivalent in design to those reported in the main text and had several significant methodological limitations (see Lind, 2010). Crucially, instead of comparing self-referential encoding to other-person-referential encoding, the researchers compared it to phonological and semantic encoding. Hence their results (a reduced self-reference effect in ASD) may be explained by the type of social processing (evaluating whether a personality trait applies to a person vs. judging the phonological properties or meaning of a trait word) required during the encoding phase or something specific to the self.

2 Data reported in the papers by Henderson et al. and Burrows et al. were overlapping. Burrows et al. reported their sample size as comprising 79 participants with ASD and 73 comparison participants. However, 31 of the participants in each group had been included in the study by Henderson et al., so the results of each study were not independent. Heather Henderson kindly provided us with the data from only those participants who took part in the study by Burrows et al., which allowed us to calculate an average weighted effect size for the between-group difference in the size of self-bias in each study independently.

3 Post hoc analyses showed the difference between men and women in implicit, *t*(255) = 0.54, *p* = .592, *d* = 0.08, and explicit, *t*(255) = 0.36, *p* = .720, *d* = 0.05, self-bias scores were negligible and nonsignificant. Moreover, the difference between men and women in the size of the correlation between AQ and implicit self-bias score, *z* = 0.31, *p* = .757, and AQ and explicit self-bias, *z* = 0.50, *p* = .617, was not significant.

4 Clearly, this lack of a significant effect was the result of a loss of statistical power from splitting groups. The effect size associated with the size of implicit self-bias in both groups together (d = 0.26) was very similar to that observed in participants with ASD (*d* = 0.31) and NT (*d* = 0.22); the effect size was very similar in each group, as well as to the overall effect size for the size of the implicit self-bias reported in the ANOVA. Moreover, breaking down groups in this way is not strictly valid, because none of the ANOVA interaction effects involving group even approached significance or were associated with effect sizes greater than negligible in magnitude (all *p*s ≥ .65; all η_*p*_^2^ ≤ .005). However, we broke down results in this way for full transparency.

## References

[c1] Abell F., Happé F., & Frith U. (2000). Do triangles play tricks? Attribution of mental states to animated shapes in normal and abnormal development. Cognitive Development, 15, 1–16. 10.1016/S0885-2014(00)00014-9

[c2] American Psychiatric Association (2000). Diagnostic and statistical manual of mental disorders (4th ed., text rev. Washington DC: Author.

[c3] Bailey A., Le Couteur A., Gottesman I., Bolton P., Simonoff E., Yuzda E., & Rutter M. (1995). Autism as a strongly genetic disorder: Evidence from a British twin study. Psychological Medicine, 25, 63–77. 10.1017/S0033291700028099 7792363

[c4] Baron-Cohen S., Wheelwright S., Hill J., Raste Y., & Plumb I. (2001). The “Reading the Mind in the Eyes” Test revised version: A study with normal adults, and adults with Asperger syndrome or high-functioning autism. Journal of Child Psychology and Psychiatry, 42, 241–251. 10.1111/1469-7610.00715 11280420

[c5] Baron-Cohen S., Wheelwright S., Skinner R., Martin J., & Clubley E. (2001). The autism-spectrum quotient (AQ): Evidence from Asperger syndrome/high-functioning autism, males and females, scientists and mathematicians. Journal of Autism and Developmental Disorders, 31, 5–17. 10.1023/A:1005653411471 11439754

[c6] Bishop D. (2011, 5 30). Are our “gold standard” autism diagnostic instruments fit for purpose? [Web log message] Retrieved from http://deevybee.blogspot.co.uk/2011_05_01_archive.html

[c7] Burrows C. A., Usher L. V., Mundy P. C., & Henderson H. A. (2017). The salience of the self: Self-referential processing and internalizing problems in children and adolescents with autism spectrum disorder. Autism Research, 10, 949–960. 10.1002/aur.1727 27868365 PMC5438783

[c9] Constantino J. N., Davis S. A., Todd R. D., Schindler M. K., Gross M. M., Brophy S. L., . . . Reich W. (2003). Validation of a brief quantitative measure of autistic traits: Comparison of the social responsiveness scale with the autism diagnostic interview-revised. Journal of Autism and Developmental Disorders, 33, 427–433. 10.1023/A:1025014929212 12959421

[c10] Constantino J. N., & Gruber C. P. (2005). Social Responsive Scale (SRS) manual. Los Angeles, CA: Western Psychological Services.

[c11] Constantino J. N., & Todd R. D. (2003). Autistic traits in the general population: A twin study. Archives of General Psychiatry, 60, 524–530. 10.1001/archpsyc.60.5.524 12742874

[c12] Conway M. A. (2005). Memory and the self. Journal of Memory and Language, 53, 594–628. 10.1016/j.jml.2005.08.005

[c13] Conway M. A., & Dewhurst S. A. (1995). The self and recollective experience. Applied Cognitive Psychology, 9, 1–19. 10.1002/acp.2350090102

[c14] Cooper R. A., Plaisted-Grant K. C., Baron-Cohen S., & Simons J. S. (2016). Reality monitoring and metamemory in adults with autism spectrum conditions. Journal of Autism and Developmental Disorders, 46, 2186–2198. 10.1007/s10803-016-2749-x 26899724 PMC4860197

[c15] Craik F. I., & Tulving E. (1975). Depth of processing and the retention of words in episodic memory. Journal of Experimental Psychology: General, 104, 268–294. 10.1037/0096-3445.104.3.268

[c16] Cunningham S. J., Brebner J. L., Quinn F., & Turk D. J. (2014). The self-reference effect on memory in early childhood. Child Development, 85, 808–823. 10.1111/cdev.12144 23888928

[c18] Dienes Z. (2014). Using Bayes to get the most out of non-significant results. Frontiers in Psychology, 5, 781 10.3389/fpsyg.2014.00781 25120503 PMC4114196

[c19] Ehlers S., Gillberg C., & Wing L. (1999). A screening questionnaire for Asperger syndrome and other high-functioning autism spectrum disorders in school age children. Journal of Autism and Developmental Disorders, 29, 129–141. 10.1023/A:1023040610384 10382133

[c20] Fanelli D. (2010). “Positive” results increase down the Hierarchy of the Sciences. PLoS ONE, 5(4), e10068 10.1371/journal.pone.0010068 20383332 PMC2850928

[c21] Faul F., Erdfelder E., Buchner A., & Lang A. G. (2009). Statistical power analyses using G*Power 3.1: Tests for correlation and regression analyses. Behavior Research Methods, 41, 1149–1160. 10.3758/BRM.41.4.1149 19897823

[c22] Frazier T. W., Ratliff K. R., Gruber C., Zhang Y., Law P. A., & Constantino J. N. (2014). Confirmatory factor analytic structure and measurement invariance of quantitative autistic traits measured by the Social Responsiveness Scale-2. Autism, 18, 31–44. 10.1177/1362361313500382 24019124

[c23] Frith U. (2003). Autism: Explaining the enigma. Malden, MA: Blackwell Publishing.

[c24] Frith U. (2012). Why we need cognitive explanations of autism. The Quarterly Journal of Experimental Psychology, 65, 2073–2092. 10.1080/17470218.2012.697178 22906000

[c25] Grainger C., Williams D. M., & Lind S. E. (2014). Online action monitoring and memory for self-performed actions in autism spectrum disorder. Journal of Autism and Developmental Disorders, 44, 1193–1206. 10.1007/s10803-013-1987-4 24193578

[c26] Grainger C., Williams D. M., & Lind S. E. (2016). Metacognitive monitoring and control processes in children with autism spectrum disorder: Diminished judgement of confidence accuracy. Consciousness and Cognition: An International Journal, 42, 65–74. 10.1016/j.concog.2016.03.003 26985883

[c27] Grisdale E., Lind S. E., Eacott M. J., & Williams D. M. (2014). Self-referential memory in autism spectrum disorder and typical development: Exploring the ownership effect. Consciousness and Cognition: An International Journal, 30, 133–141. 10.1016/j.concog.2014.08.023 25286242

[c28] Heider F., & Simmel M. (1944). An experimental study of apparent behavior. The American Journal of Psychology, 57, 243–259. 10.2307/1416950

[c29] Henderson H. A., Zahka N. E., Kojkowski N. M., Inge A. P., Schwartz C. B., Hileman C. M., . . . Mundy P. C. (2009). Self-referenced memory, social cognition, and symptom presentation in autism. Journal of Child Psychology and Psychiatry, 50, 853–861. 10.1111/j.1469-7610.2008.02059.x 19298471 PMC2697280

[c30] Hobson R. P. (1990). On the origins of self and the case of autism. Development and Psychopathology, 2, 163–181. 10.1017/S0954579400000687

[c31] Hobson R. P. (2010). Explaining autism: Ten reasons to focus on the developing self. Autism, 14, 391–407. 10.1177/1362361310364142 20926456

[c32] Hume D. (2003). A treatise of human nature. London, UK: Dent (Original work published 1739)

[c33] James W. (1890). The principles of psychology. New York, NY: Holt.

[c34] JASP Team (2016). JASP (Version 0.8.1) [Computer software]. Retrieved from https://jasp-stats.org/

[c35] Jeffreys H. (1961). Theory of probability (3rd ed.). Oxford, UK: Oxford University Press.

[c36] Klein S. B., & Kihlstrom J. F. (1986). Elaboration, organization, and the self-reference effect in memory. Journal of Experimental Psychology: General, 115, 26–38. 10.1037/0096-3445.115.1.26 2937872

[c37] Klein S. B., & Loftus J. (1988). The nature of self-referent encoding: The contributions of elaborative and organizational processes. Journal of Personality and Social Psychology, 55, 5–11. 10.1037/0022-3514.55.1.5

[c38] Kühberger A., Fritz A., & Scherndl T. (2014). Publication bias in psychology: A diagnosis based on the correlation between effect size and sample size. PLoS ONE, 9(9), e105825 10.1371/journal.pone.0105825 25192357 PMC4156299

[c39] Kuiper N. A. (1982). Processing personal information about well-known others and the self: The use of efficient cognitive schemata. Canadian Journal of Behavioural Science/Revue canadienne des sciences du comportement, 14, 1–12.

[c40] Lind S. E. (2010). Memory and the self in autism: A review and theoretical framework. Autism, 14, 430–456. 10.1177/1362361309358700 20671017

[c41] Lind S. E., Williams D. M., Grainger C., & Landsiedel J. (2018). The self in autism and its relation to memory In Johnson J. L., Goodman G. S., & Mundy P. C. (Eds.), The Wiley handbook of memory, autism spectrum disorder, and the law (pp. 70–91). Hoboken, NJ: John Wiley & Sons Ltd.

[c42] Locke J. (1995). An essay concerning human understanding. New York, NY: Prometheus (Original work published 1690)

[c43] Lombardo M. V., Barnes J. L., Wheelwright S. J., & Baron-Cohen S. (2007). Self-referential cognition and empathy in autism. PLoS ONE, 2(9), e883 10.1371/journal.pone.0000883 17849012 PMC1964804

[c44] Lombardo M. V., & Baron-Cohen S. (2010). Unraveling the paradox of the autistic self. Wiley Interdisciplinary Reviews: Cognitive Science, 1, 393–403. 10.1002/wcs.45 26271379

[c45] Lord C., Risi S., Lambrecht L., Cook E. H. Jr., Leventhal B. L., DiLavore P. C., . . . Rutter M. (2000). The autism diagnostic observation schedule-generic: A standard measure of social and communication deficits associated with the spectrum of autism. Journal of Autism and Developmental Disorders, 30, 205–223. 10.1023/A:1005592401947 11055457

[c46] Mandy W., Chilvers R., Chowdhury U., Salter G., Seigal A., & Skuse D. (2012). Sex differences in autism spectrum disorder: Evidence from a large sample of children and adolescents. Journal of Autism and Developmental Disorders, 42, 1304–1313. 10.1007/s10803-011-1356-0 21947663

[c47] Masicampo E. J., & Lalande D. R. (2012). A peculiar prevalence of p values just below. 05. The Quarterly Journal of Experimental Psychology, 65, 2271–2279. 10.1080/17470218.2012.711335 22853650

[c48] Miller G. A., & Chapman J. P. (2001). Misunderstanding analysis of covariance. Journal of Abnormal Psychology, 110, 40–48. 10.1037/0021-843X.110.1.40 11261398

[c49] Millward C., Powell S., Messer D., & Jordan R. (2000). Recall for self and other in autism: Children’s memory for events experienced by themselves and their peers. Journal of Autism and Developmental Disorders, 30, 15–28. 10.1023/A:1005455926727 10819117

[c50] Nicholson T., Williams D. M., Grainger C., Lind S. E., & Carruthers P. (2019). Relationships between implicit and explicit uncertainty monitoring and mindreading: Evidence from autism spectrum disorder. Consciousness and Cognition: An International Journal, 70, 11–24. 10.1016/j.concog.2019.01.013 30776592

[c51] Peterson C. C., Wellman H. M., & Liu D. (2005). Steps in theory-of-mind development for children with deafness or autism. Child Development, 76, 502–517. 10.1111/j.1467-8624.2005.00859.x 15784096

[c52] Powell S. D., & Jordan R. R. (1996). Understanding memory in autism. International Journal of Psychology, 31, 440.2.

[c53] Prebble S. C., Addis D. R., & Tippett L. J. (2013). Autobiographical memory and sense of self. Psychological Bulletin, 139, 815–840. 10.1037/a0030146 23025923

[c54] Risi S., Lord C., Gotham K., Corsello C., Chrysler C., Szatmari P., . . . Pickles A. (2006). Combining information from multiple sources in the diagnosis of autism spectrum disorders. Journal of the American Academy of Child & Adolescent Psychiatry, 45, 1094–1103. 10.1097/01.chi.0000227880.42780.0e 16926617

[c55] Rogers T. B., Kuiper N. A., & Kirker W. S. (1977). Self-reference and the encoding of personal information. Journal of Personality and Social Psychology, 35, 677–688. 10.1037/0022-3514.35.9.677 909043

[c56] Rosenthal R. (1979). The file drawer problem and tolerance for null results. Psychological Bulletin, 86, 638–641. 10.1037/0033-2909.86.3.638

[c57] Rouder J. N., Speckman P. L., Sun D., Morey R. D., & Iverson G. (2009). Bayesian *t* tests for accepting and rejecting the null hypothesis. Psychonomic Bulletin & Review, 16, 225–237. 10.3758/PBR.16.2.225 19293088

[c58] Russell J. (1996). Agency: Its role in mental development. Hove, UK: Psychology Press.

[c59] Schmitz T. W., & Johnson S. C. (2007). Relevance to self: A brief review and framework of neural systems underlying appraisal. Neuroscience and Biobehavioral Reviews, 31, 585–596. 10.1016/j.neubiorev.2006.12.003 17418416 PMC1973156

[c60] Sui J., & Humphreys G. W. (2015). The integrative self: How self-reference integrates perception and memory. Trends in Cognitive Sciences, 19, 719–728. 10.1016/j.tics.2015.08.015 26447060

[c61] Sui J., & Humphreys G. W. (2017). The self survives extinction: Self-association biases attention in patients with visual extinction. Cortex: A Journal Devoted to the Study of the Nervous System and Behavior, 95, 248–256. 10.1016/j.cortex.2017.08.006 28922647

[c62] Symons C. S., & Johnson B. T. (1997). The self-reference effect in memory: A meta-analysis. Psychological Bulletin, 121, 371–394. 10.1037/0033-2909.121.3.371 9136641

[c63] Toichi M., Kamio Y., Okada T., Sakihama M., Youngstrom E. A., Findling R. L., & Yamamoto K. (2002). A lack of self-consciousness in autism. The American Journal of Psychiatry, 159, 1422–1424. 10.1176/appi.ajp.159.8.1422 12153838

[c64] Turk D. J., Cunningham S. J., & Macrae C. N. (2008). Self-memory biases in explicit and incidental encoding of trait adjectives. Consciousness and Cognition: An International Journal, 17, 1040–1045. 10.1016/j.concog.2008.02.004 18395467

[c65] Turk D. J., van Bussel K., Waiter G. D., & Macrae C. N. (2011). Mine and me: Exploring the neural basis of object ownership. Journal of Cognitive Neuroscience, 23, 3657–3668. 10.1162/jocn_a_00042 21557652

[c66] Wechsler D. (1999). Wechsler Abbreviated Scale of Intelligence. New York, NY: The Psychological Corporation: Harcourt Brace & Company.

[c67] Williams D. (2010). Theory of own mind in autism: Evidence of a specific deficit in self-awareness? Autism, 14, 474–494. 10.1177/1362361310366314 20926458

[c68] Williams D., Bergström Z., & Grainger C. (2016). Metacognitive monitoring and the hypercorrection effect in autism and the general population: Relation to autism(-like) traits and mindreading. Autism: An International Journal of Research and Practise, 22, 259–270. 10.1177/1362361316680178 29671645

[c69] Williams D. M., & Bowler D. M. (2014). Autism spectrum disorder: Fractionable or coherent? Autism, 18, 2–5. 10.1177/1362361313513523 24505609

[c70] Williams D. M., Nicholson T., & Grainger C. (2018). The self-reference effect on perception: Undiminished in adults with autism and no relation to autism traits. Autism Research, 11, 331–341. 10.1002/aur.1891 29160023 PMC5836899

[c71] Williams D. M., Peng C., & Wallace G. L. (2016). Verbal thinking and inner speech use in autism spectrum disorder. Neuropsychology Review, 26, 394–419. 10.1007/s11065-016-9328-y 27632384

[c72] Wilson A. E., & Ross M. (2003). The identity function of autobiographical memory: Time is on our side. Memory, 11, 137–149. 10.1080/741938210 12820827

[c73] Woodbury-Smith M. R., Robinson J., Wheelwright S., & Baron-Cohen S. (2005). Screening adults for Asperger Syndrome using the AQ: A preliminary study of its diagnostic validity in clinical practice. Journal of Autism and Developmental Disorders, 35, 331–335. 10.1007/s10803-005-3300-7 16119474

[c74] World Health Organization (1993). International classification of mental and behavioral disorders: Clinical descriptions and diagnostic guidelines (10th ed.). Geneva, Switzerland: Author.

