# Supplementary material for: The Self-Reference Effect on Memory Is Not Diminished in Autism: Three Studies of Incidental and Explicit Self-Referential Recognition Memory in Autistic and Neurotypical Adults and Adolescents
Source: J Abnorm Psychol. 2019 Oct 31;129(2):224–36. doi: 10.1037/abn0000467 (PMC6975523; doi:10.1037/abn0000467)
Supplement: Supplementary file 1 [file abn0000467_Suppl.docx]

**Part 1: Supplementary Material for Experiment 1**

**Supplementary Table 1**

Hit rates (proportions of old items corrected identified as old), false alarm rates (proportions of new items incorrectly identified as old) for each condition and referent.

|  |  | Hit rate | |  | False alarm rate* | |  |
| --- | --- | --- | --- | --- | --- | --- | --- |
| Condition | Referent | *M (SD)* | Range |  | *M (SD)* | Range |  |
| Implicit | Self | .66 (.17) | 0.04-1.00 |  | .19 (.13) | 0.00-0.67 |  |
|  | Other | .61 (.16) | 0.10-1.00 |  |  |  |  |
| Explicit | Self | .88 (.14) | 0.17-1.00 |  | .14 (13) | 0.00-0.83 |  |
|  | Other | .79 (.16) | 0.13-1.00 |  |  |  |  |

*Note.* False alarm rates were independent of referent (false alarms were incorrect “yeses” during the test phase and were never presented in relation to self or other)

To ensure that floor or ceiling effects had not occurred in the self-referential memory task, a series of one-sample t-tests were carried out on the hit rates reported above. These revealed that all hit rates were significantly greater than 0 (the floor score), all *t*s ≥ 59.06, all *p*s < .001, and significantly less than 1 (the ceiling score), all *t*s (256) ≥ -13.71=, all *p*s < .001.

**Supplementary Figure 1**

Scatterplots showing the relation between Autism-spectrum Quotient (AQ) score and explicit (top) and implicit (bottom) self bias score (data from Experiment 1)

**
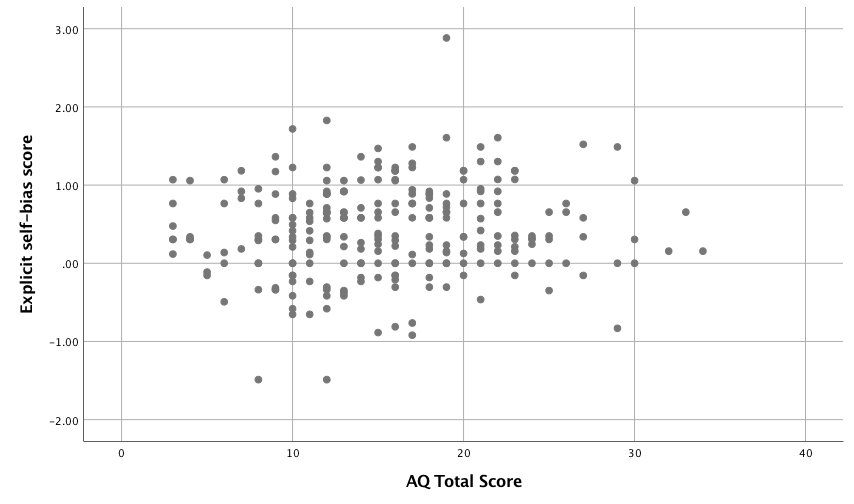
**

**
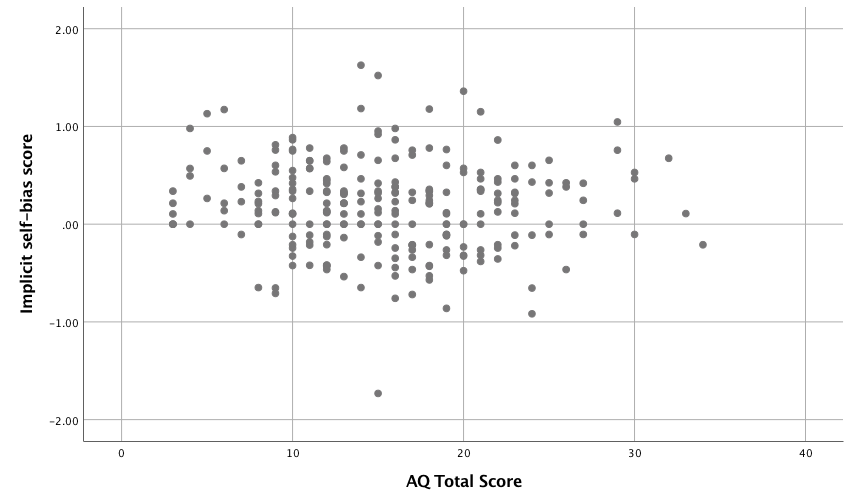
**

**Part 2: Supplementary Material for Experiment 2**

**a) Methods for Mindreading Tasks**

**Reading the Mind in the Eyes.**

Reading the Mind in the Eyes (Baron-Cohen, Wheelwright, Hill, Raste & Plumb, 2001) is a widely used measure of mindreading. Participants were presented with a series of 36 photographs of the eye-region of the face. On each trial, participants were asked to pick one word from a selection of four to indicate what the person in the picture was thinking/feeling. Scores ranged from a possible 0–36, with higher scores indicating better performance. Proportion correct (*n*/36) scores for each group are presented in Table 2.

**Animations task.**

The Animations task, which is based on Heider and Simmel (1944), required participants to describe interactions between a large red triangle and a small blue triangle, as portrayed in a series of silent video clips (Abell, Happe & Frith, 2000). Four clips (out of 12) were apt to invoke an explanation of the triangles’ behavior in terms of epistemic mental states, such as belief, intention, and deception. These clips comprise the “mentalizing” condition of the task and were employed in this study. Each clip was presented to participants on a computer screen. After the clip was finished, participants described what had happened in the clip. An audio recording of participants’ responses was made for later transcription. Each transcription was scored on a scale of 0–2 for accuracy, based on the criteria outlined in Abell et al. (2000). Seventy-five percent of transcripts were also scored by two independent raters. Inter-rater reliability across all clips was excellent according to Cicchetti’s (1994) criteria (intra-class correlation = .86). Accuracy (proportion: *n*/8) among ASD and comparison participants is shown in Table 2.

**b) Experiment 2 Subsample Analyses**

After excluding participants with ASD who scored under the cut-offs (or who had missing data) on the ADOS or AQ, and NT participants who scored over the cut-off on the AQ, we were left with 19 participants with ASD and 29 NT participants (these reduced samples remained matched on sex, age, VIQ, PIQ and FSIQ). Using these subsamples, we conducted a 2 (Condition: implicit/explicit) × 2 (Referent: self/other) × 2 (Group: ASD/NT) mixed ANOVA on d’ scores. Descriptive and inferential statistics are reported in Supplementary Tables 2 and 3. Both main effects of Referent and Condition were significant, and the interaction between them was near to statistical significance (*p* = .080) and indicated self-bias in both conditions, but larger in the explicit than implicit condition). None of the effects involving Group approached significance.

**Supplementary Table 2**

*Experiment 2 Descriptive Statistics for d’ (Recognition Memory Accuracy) Measures in Each Condition Among ASD and Neurotypical Participants after excluding participants with ASD who scored under the cut-offs on the ADOS or AQ, and NT participants who scored over cut-off on the AQ*

|  |  | ASD subsample (*n* = 19) | |  | NT subsample (*n* = 29) | |  | Total subsample (*n* = 58) | |
| --- | --- | --- | --- | --- | --- | --- | --- | --- | --- |
| Condition | Measure | *M (SD)* | Range |  | *M (SD)* | Range |  | *M (SD)* | Range |
| Implicit | Self d’ | 1.48 (0.62) | 0.16-2.88 |  | 1.20 (0.74) | -0.21-2.85 |  | 1.31 (0.70) | -0.21-2.88 |
|  | Other d’ | 1.29 (0.65) | 0.54-2.70 |  | 1.00 (0.68) | -0.29-2.14 |  | 1.11 (0.68) | -0.29-2.70 |
|  | Self-bias | 0.19 (0.52) | -1.06-1.49 |  | 0.20 (0.43) | -0.78-1.14 |  | 0.20 (0.46) | -1.06-1.49 |
| Explicit | Self d’ | 2.40 (0.64) | 1.52-3.46 |  | 2.33 (0.74) | 0.97-3.77 |  | 2.36 (0.69) | 0.97-3.77 |
|  | Other d’ | 1.89 (0.84) | 0.42-3.42 |  | 2.04 (0.56) | 1.11-3.46 |  | 1.98 (0.68) | 0.42-3.46 |
|  | Self-bias | 0.50 (0.62) | -0.58-1.41 |  | 0.29 (0.59) | -0.76-1.30 |  | 0.38 (0.60) | -0.76-1.41 |

*Note.* Self bias score = self d’ minus other d’. ASD = autism spectrum disorder; NT = neurotypical

**Supplementary Table 3**

*ANOVA results from Experiment 2 (dependent variable = d’ score) after excluding participants with ASD who scored under the cut-offs on the ADOS or AQ and NT participants who scored over cut-off on the AQ*

| Variable | *F*(1,46) | *p* | $\eta_{p}^{2}$ | Direction of effect |
| --- | --- | --- | --- | --- |
| Condition | 59.09 | **< .001** | .56 | Explicit > Implicit |
| Referent | 28.24 | **<.001** | .38 | Self > Other |
| Group | 0.73 | .396 | .02 | - |
| Condition × Referent | 3.21 | .080 | .07 | - |
| Condition × Group | 1.88 | .177 | .04 | - |
| Referent × Group | 0.88 | .360 | .02 | - |
| Condition × Group × Referent | 0.85 | .361 | .02 | - |

There was no significant between-group difference in the size of the self-reference effect (difference between self d’ and other d’) in either the explicit condition, *t*(46) = 1.17, *p* = .993, *d* < 0.01, BF^10^ = 0.51 (note, size of self-bias numerically larger in ASD than NT group), or implicit condition, *t*(46) < 0.01, *p* = .993, *d* < 0.01, BF^10^ = 0.29. Among participants with ASD, the self-bias was significant in the explicit condition, *t*(18)= 3.54, *p* = .002, *d* = 0.81, BF^10^ 17.72, but not implicit condition, *t*(18)= 1.63, *p* = .121, *d* = 0.37, BF^10^ = 0.73. Among participants with ASD, the self-bias was significant in the explicit condition, *t*(28)= 2.68, *p* = .012, *d* = 0.50, BF^10^ = 4.98, and the implicit condition, *t*(28)= 2.45, *p* = .021, *d* = 0.46, BF^10^ = 2.48.

**Part 3: Supplementary Material for Experiment 3**

**a) Method Details for Mindreading Measures.**

Participants completed the same mindreading measures used in Experiment 2, although the RMIE used was the adapted child version of the RMIE (Baron-Cohen, Wheelwright, Spong, Scahill & Lawson, 2001). Seventy-five percent of Animations transcripts were scored by two independent raters. Inter-rater reliability was excellent according to Cicchetti’s [1994] criteria (intra-class correlation = .85). Accuracy (proportion) on the RMIE and Animations tasks among ASD and comparison participants is shown in Table 5.

**b) Experiment 3 Subsample Analyses**

After excluding participants with ASD who scored under, and NT participants who scored over, the cut-off on the SRS, we were left with groups of 25 and 24, respectively (the reduced sub-samples remained matched on sex, age, VIQ, PIQ and FSIQ). Using these subsamples, we conducted a 2 (Condition: implicit/explicit) × 2 (Referent: self/other) × 2 (Group: ASD/NT) mixed ANOVA on d’ scores. Descriptive and inferential statistics are reported in Supplementary Tables 4 and 5. None of the ANOVA effects involving Group even approached significance.

**Supplementary Table 4**

*Experiment 3 Descriptive Statistics for d’ (Recognition Memory Accuracy) Measures in Each Condition Among ASD and Neurotypical Participants*, after excluding participants whose SRS scores were discrepant with their diagnostic status

|  |  | ASD (*n* = 25) | |  | NT subsample (*n* = 24) | |  | Total subsample (*N* = 49) | |
| --- | --- | --- | --- | --- | --- | --- | --- | --- | --- |
| Condition | Measure | *M (SD)* | Range |  | *M (SD)* | Range |  | *M (SD)* | Range |
| Implicit | Self d’ | 2.04 (0.69) | 0.95-3.78 |  | 2.05 (0.73) | 0.60 -3.13 |  | 2.04 (0.70) | 0.60-3.38 |
|  | Other d’ | 1.93 (0.60) | 0.71-3.13 |  | 2.00 (0.75) | 0.50-3.08 |  | 1.96 (0.67) | 0.50-3.13 |
|  | Self-bias | 0.11 (0.45) | -0.84-0.96 |  | 0.05 (0.58) | -1.19-1.19 |  | 0.08 (0.51) | -1.19-1.19 |
| Explicit | Self d’ | 2.67 (0.73) | 1.19-3.78 |  | 2.77 (0.74) | 0.50-3.78 |  | 2.72 (0.73) | 0.50-3.78 |
|  | Other d’ | 2.29 (0.58) | 0.81-3.45 |  | 2.51 (0.76) | 0.71-3.78 |  | 2.40 (0.68) | 0.71-3.78 |
|  | Self-bias | 0.38 (0.54) | -0.96-1.35 |  | 0.26 (0.69) | -0.96-3.78 |  | 0.32 (0.61) | -0.96-1.51 |

*Note.* Self bias score = self d’ minus other d’. ASD = autism spectrum disorder; NT = neurotypical

**Supplementary Table 5**

ANOVA results from Experiment 3, after excluding participants whose SRS scores were discrepant with their diagnostic status

| Variable | *F(47)* | *p* | $\eta_{p}^{2}$ | Direction of effect |
| --- | --- | --- | --- | --- |
| Condition | 23.85 | **< .001** | .34 | Explicit > Implicit |
| Referent | 13.38 | **<.001** | .22 | Self > Other |
| Group | 0.48 | .494 | .01 | - |
| Condition × Referent | 4.06 | .050 | .08 | - |
| Condition × Group | 0.28 | .599 | <.01 | - |
| Referent × Group | 0.67 | .419 | .01 | - |
| Condition × Group × Referent | 0.06 | .807 | <.01 | - |

There was no significant between-group difference in the size of the self-bias in either the explicit condition, *t*(47) = 0.68, *p* = .502, *d* = 0.19, BF^10^ = 0.34, or implicit condition, *t*(47) = 0.41, *p* = .688, *d* = 0.12, BF^10^ = 0.31. The self-reference effect in the explicit condition was significant among participants with ASD, *t*(24) = 3.54, *p* = .002, *d* = 0.71, BF^10^ = 22.31, and NT participants, *t*(23) = 1.87, *p* = .037 (one-tailed), *d* = 0.38, BF^10^ = 0.95. When the self-reference effect in the implicit condition was analysed in each group separately, it was non-significant in either participants with ASD, *t*(24) = 1.21, *p* =.237, *d* = 0.24, BF^10^ = 0.41, or NT participants, *t*(23) = 0.42, *p* = .677, *d* = 0.09, BF^10^ = 0.23.
